# Identification of resistance to stripe rust in 267 Chinese spring wheat landraces germplasm and molecular detection of disease resistance genes

**DOI:** 10.1016/j.bbrep.2025.102206

**Published:** 2025-08-17

**Authors:** Miaomiao Huang, Yuqing Zhang, Wanwei Hou, Ping Jin, Liang Huang

**Affiliations:** a Academy of Agricultural and Forestry Sciences, Qinghai University, Xining, Qinghai, 810016, China; b State Key Laboratory for the Biology of Plant Diseases and Insect Pests, Institute of Plant Protection, Chinese Academy of Agricultural Science, Beijing, 100193, China; c National Crop Germplasm Resources Duplicate, Xining, Qinghai, 810016, China; d National Agricultural Experimental Station for Plant Protection, Gangu, Ministry of Agriculture and Rural Affairs, Gansu, 741200, China

**Keywords:** Spring wheat landraces, Stripe rust, Resistance identification, *Yr*

## Abstract

Stripe rust (*Puccinia striiformis* f. sp. *tritici*) poses a major threat to Chinese wheat production. Assessing resistance levels and gene distribution in spring wheat landraces is critical for breeding durable resistant cultivars. This study evaluated 267 landraces from eight provinces for seedling resistance against dominant races CYR32/CYR34 and adult-plant resistance (APR) across five Qinghai natural disease nurseries during 2023–2024. At the seedling stage, 7.5 % (20 accessions; IT 0–6) and 12.4 % (33; IT 0–6) exhibited resistance to CYR32 and CYR34, respectively, with 2.99 % (8 accessions) conferring dual resistance. APR trials identified2.25 % (6 landraces) showed stable resistance, including Banjiemang with complete immunity (IT 0). Molecular analyses revealed prevalent *Yr* genes, *Yr10* (38.6 %,103/267), *Yr18* (29.6 %,79/267), *Yr26* (20.9 %, 56/267) and multi-gene combinations 60 accessions (22.5 %) with 2 genes; 33 (12.4 %) with ≥3 genes. Notably, 97 accessions (36.3 %) lacked known *Yr* genes, suggesting novel resistance mechanisms. The *Yr18/Yr2*6 combination emerged as a promising APR source. This study provides germplasm and genetic insights for pyramiding strategies to enhance stripe rust resistance.

## Highlights

• 7.5 % & 12.4 % of 267 Chinese spring wheat landraces resist CYR32/CYR34, 3.4 % dual-resistant.
• 2.25 % landraces show adult-plant resistance; Banjiemang (Gandi 816) completely immune.
• *Yr5*, *Yr9*, *Yr10* genes detected; 60 accessions with two-gene, 33 multi-gene combos.
• 36.3 % accessions lack known *Yr* genes, indicating possible novel resistance mechanisms.
• *Yr18*/*Yr26* combos identified as key APR sources for durable rust-resistant wheat.

## 1 Introduction

Stripe rust, caused by *Puccinia striiformis* f. sp. *tritici* (*Pst*), represents a major biotic constraint to achieving sustainable wheat productivity and global food security [1,2]. In China, yield losses attributable to *Pst* infection range from 20 to 30 % under epidemic conditions, escalating to more than 50 % (occasionally 100 %) during severe epidemic years [3]. Practice has proven that breeding and planting resistant cultivars is the most economical, environmentally friendly, and effective measure to control the disease. Screening of resistant germplasm resources and exploration of resistance genes are the foundation of disease-resistant breeding. To date, over 300 resistance genes/QTLs (designated *Yr1* to *Yr87*) have been identified across all 21 chromosomes (1A–7D) of wheat have been reported [4]. Nevertheless, there are still many contradictions, such as the high frequency of virulence variation in *Pst* races, slow breeding speed of resistant cultivars, and the limited application of *Yr* genes in wheat breeding despite their extensive exploration [5]. So far, only a few *Yr* genes, such as *Yr5* and *Yr15*, remain effective against all known *Pst* races globally [6,7]. In China, a new *Pst* race TSA-6, which was first identified in Baoji, Shaanxi Province, can infect cultivars harboring *Yr5* resistance [[8], [9], [10]]. Additionally, non-race-specific adult-plant resistance (APR) genes like *Yr18*, which confers partial field resistance to stripe rust, leaf rust, stem rust, and powdery mildew have been used in breeding for a century with no observed pathogen adaptation [11]. Furthermore, the extensive use of a single resistance gene and the continuous variation of pathogen toxicity can lead to the overcoming of resistance genes by new virulent races of the pathogen, resulting in the “loss” of variety resistance and causing new disease epidemics, posing a severe threat to grain production.

Of particular agricultural significance are landrace cultivars-genetic resources shaped by millennia of farmer selection and environmental adaptation. These accessions demonstrate exceptional ecological plasticity, stable yield performance under marginal conditions, and allelic diversity absent in modern cultivars, including uncharacterized disease resistance determinants [12,13]. Chinese wheat landraces exhibit unique agronomic traits such as photoperiod insensitivity, multi-spikelet architecture, broad adaptation, and combined biotic/abiotic stress resilience, making them invaluable for genetic improvement programs [14]. Particularly in the resistance to wheat stripe rust, systematic research has been conducted on Chinese landrace wheat, ranging from resource identification, resistance gene mining, to breeding utilization. In recent years, with the emergence and prevalence of new highly virulent races of stripe rust, it has become a consensus to mine new effective resistance genes from landrace wheat and cultivate new varieties resistant to stripe rust. Pedigree analysis has revealed that the four officially named new genes for wheat stripe rust resistance, *Yr79* [15], *Yr80* [16], *Yr81* [17], and *Yr82* [18], recently reported, all originate from landrace wheat in different countries, demonstrating that wheat landrace carry a rich array of useable stripe rust resistance genes and are an effective gene source for wheat stripe rust resistance breeding. However, there is currently a lack of systematic research on spring wheat landrace germplasm resources, especially in the Qinghai-Tibet Plateau region, concerning the identification of resistance to stripe rust and the mining of resistance genes in spring wheat landrace germplasm resources.

The epidemic of stripe rust in China is divided into four major epidemic areas and five oversummering regions of the *Pst*. Regardless of the area of inoculum sources or the amount of inoculum provided, the northwest region (Gansu, Qinghai and Ningxia) is the most important oversummering area [2,3]. Successful *Pst* oversummering requires continuous host-pathogen coexistence [19]. The western regions are mostly mixed planting areas of winter and spring wheat, where the *Pst* can oversummering through continuous infection by urediniospores at altitudes above 1800 m, especially above 2000 m, on self-sown wheat seedlings and late-maturing spring wheat in mountain slopes and plateau areas [20]. In autumn, the oversummering inoculum is spread to the plain winter wheat areas and lower altitude winter wheat areas by northwest air currents, infecting and harming the autumn-sown wheat seedlings [21]. In Qinghai Province, wheat is planted at altitudes ranging from 1700 to 3200 m, with late-maturing spring wheat (maturing through October) and volunteer wheat of winter wheat emerging from late July. From late July to early September, the late-maturing spring wheat and volunteer wheat coexist and overlap in their growth period for more than 40 days, providing a large amount of oversummering inoculum, which continues until the emergence of early-sown winter wheat. Almost all wheat planting areas in the province are suitable for the oversummering of *Pst* [22, 23], which is very conducive to the continuous reproduction and transfer infection of the *Pst*. Therefore, spring wheat is an important host for the oversummering of *Pst* in the western region. However, the resistance of spring wheat plants in the cold highland areas of the west has been generally lost [24]. Previous systematic studies on the identification and resistance gene mining of wheat landrace in the Qinghai-Tibet Plateau region is relatively few, and there is insufficient research on local special germplasm resources resistant to stripe rust.

The northwest region of China is an important source of *Pst*, and some areas are important transit areas for the epidemic of stripe rust. Therefore, understanding the resistance level of spring wheat to stripe rust in the northwest region is of great significance for the integrated control of wheat stripe rust in China. Selecting spring wheat cultivars with good resistance is of great importance for the prevention and control of stripe rust in the western region and even in China. Continuously mining new resistance genes from wheat cultivars (lines), exogenous materials, and landrace cultivars, tracking target genes through molecular markers, achieving precise breeding, accelerating breeding progress, and aggregating multiple types of resistance genes to cultivate high-level durable resistant cultivars are of great significance for the long-term and effective control of wheat stripe rust. Therefore, this study conducted seedling and adult plant resistance and molecular marker detection of *Yr* genes in 267 spring wheat landraces germplasm resources from 8 provinces (Qinghai, Xizang, Gansu, Sichuan, and Yunnan et al.) in China, in order to quickly clarify the resistance level and types of resistance genes carried by existing spring wheat landrace germplasm resources, providing a basis for wheat breeding in the western region.

## 2 Materials and methods

### 2.1 Experimental materials

**Test Materials:** 267 spring wheat landrace germplasm resources were sourced from Qinghai, Xizang, Sichuan, Yunnan, Inner Mongolia, Ningxia, Xinjiang, and Gansu, totaling 8 provinces (Table 1, Table S1), and were provided by the Agricultural and Forestry Science Academy of Qinghai University. Eight positive control materials: Avocet S∗6/*Yr5*、Avocet S∗6/*Yr9*、Avocet S∗6/*Yr10*、Avocet S∗6/*Yr15*、Avocet S∗6/*Yr17*、Avocet S∗6/*Yr18*、Avocet S∗6/*Yr26* and Avocet S∗6/*YrSP*, and negative control Avocet S, were all provided by Professor Xianming Chen from Washington State University. The susceptible control Mingxian 169 was provided by the Institute of Plant Protection, Chinese Academy of Agricultural Sciences (IPPCAAS).

**Table 1:** Source and number of 267 spring wheat landrace germplasm resources.

Province	Number	Province	Number
Qinghai	34	Yunnan	42
Xizang	28	Ningxia	12
Sichuan	32	Xinjiang	8
Inner Mongolia	14	Gansu	97

***Pst* races:** Two *Pst* races (CYR32 and CYR34), were used for indoor seedling stripe rust resistance evaluation, provided by the Cereal Disease Group of the Institute of Plant Protection, Chinese Academy of Agricultural Sciences (IPPCAAS).

### 2.2 Seedling resistance phenotype identification

The seedling stripe rust phenotype identification of 267 spring wheat landraces was completed in the greenhouse of the Institute of Plant Protection, Chinese Academy of Agricultural Sciences. The wheat cultivars to be tested and the susceptible controls Mingxian 169 and Avocet S were sequentially numbered and sown in 4 cm × 20 cm × 15 cm seedling boxes, with 7–9 seeds per variety. When the wheat seedlings reached the one-leaf and one-heart stage, fresh urediniospores of CYR32 and CYR34 were mixed with mineral oil Soltrol® 170 at a ratio of 50 mg/mL to prepare a 1 mL urediniospores suspension. Artificial spray inoculation was conducted according to the method of Chen et al. [25] After the urediniospores suspension on the leaves dried for 3 h, they were placed in a dark room at 10 °C for 24 h, then transferred to an artificial climate chamber with a day temperature of 15∼18 °C and a night temperature of 11∼15 °C, a photoperiod of 14 h/day, and a light intensity of 5000∼6000 lx for cultivation. Approximately 13–18 days after inoculation when rust was fully developed on Mingxian 169 and Avocet S, the infection types (ITs) were recorded based on a 0 to 9 scale [26]. Plants with IT 0 to 6 were considered resistant, whereas those with IT 7 to 9 were considered susceptible.

### 2.3 Adult plant resistance identification

Natural stripe rust infection and adult plant resistance identification were conducted in Xining City (23XN) and Huangzhong District (23HZ) of Qinghai Province in 2023, and in Xining City (24XN), Huangzhong District (24HZ), and Gonghe County (24 GH) in 2024, over two consecutive years. The sowing time for the identification plots was in early April, with each spring wheat landrace planted in a row, each row being 1 m long with a row spacing of 0.33 m. A susceptible control row of Mingxian 169 was planted every 20 cultivars, and a row of Mingxian 169 was planted perpendicularly between two cultivars as an induction row. In early July each year, under natural disease conditions (the identification plots in Xining City, Huangzhong District, and Gonghe County of Qinghai Province are common rust disease areas), when Mingxian 169 was fully diseased, the infection type (IT), severity (S), and incidence (I) of the disease were investigated. Resistance evaluation was recorded according to the national standard for wheat stripe rust resistance evaluation, recording IT of stripe rust on a scale of 0–4. Accession with IT 0–2 was categorized into the resistant and those with IT 3–4 into susceptible [27]. A total of three surveys were conducted during the wheat growth period, with the most severe reaction type and severity taken as the final resistance result.

### 2.4 Molecular marker tests

Fresh leaves were taken during the two-leaf stage of wheat, and wheat leaf DNA was extracted using the Plant Genomic DNA Kit (DP305, TianGen, China). DNA concentration was measured using the DS11+Denovix ultra-micro spectrophotometer (Thermo Fisher Scientific, Waltham, MA) and diluted to 50 ng/L with ddH_2_O. Functional markers or closely linked molecular markers for *Yr5* [28], *Yr9* [29], *Yr10* [30], *Yr15* [31], *Yr17* [32], *Yr18* [33], *Yr26* [34] and *YrSP* [35] were used to detect the 267 spring wheat materials. Primers (Table 2) were synthesized by Beijing Qingke Biotechnology Co., Ltd. Positive controls were Avocet S∗6/*Yr5*, Avocet S∗6/*Yr9*, Avocet S∗6/*Yr10*, Avocet S∗6/*Yr15*, Avocet S∗6/*Yr17*, Avocet S∗6/*Yr18*, Avocet S∗6/*Yr26* and Avocet S∗6/*YrSP*, while Mingxian 169 and Avocet S were used as negative controls. PCR amplification was performed using a PCR machine (TransGen Biotech, Beijing, China), and the results were detected using the WSE-5200 integrated gel imaging system (Atto Corporation, Tokyo, Japan) for agarose gel electrophoresis. The PCR amplification used a 25 μL reaction system, including 12.5 μL EasyTaq® PCR SuperMix, 1 μL each of upstream and downstream primers, 1 μL DNA template, and 9.5 μL ddH_2_O. The reaction program for *Yr5, Yr10* and *Yr18* was: pre-denaturation at 94 °C for 4 min; denaturation at 94 °C for 30 s, annealing at the corresponding temperature for 30s, extension at 72 °C for 30s, for a total of 35 cycles; final extension at 72 °C for 10 min. The reaction program for *Yr9* and *Yr15* was: pre-denaturation at 95 °C for 5 min; denaturation at 95 °C for 45s, annealing at the corresponding temperature for 45s, extension at 72 °C for 45s, for a total of 35 cycles; final extension at 72 °C for 10 min. The reaction program for *Yr17, Yr26* and *YrSP* was: pre-denaturation at 94 °C for 4 min; denaturation at 94 °C for 45s, annealing at the corresponding temperature for 45s, extension at 72 °C for 45s, for a total of 35 cycles; final extension at 72 °C for 10 min.

**Table 2:** Specific primer sequences and PCR programs of wheat stripe rust resistance genes.

Gene	Primer name	Primer sequence (5′-3′)	Annealing	Reference
*Yr5*	S19M93-100-F	TAATTGGGACCGAGAGACG	62 °C	[28]
	S19M93-100-R	TTCTTGCAGCTCCAAAACCT		
*Yr9*	AF1/4-F	GGAGACATCATGAAACATTTG	64 °C	[29]
	AF1/4-R	CTGTTGTTGGGCAGAAAG		
*Yr10*	Yr10-RT-F	CACTTGAGGTATCTGAGTCTAG	60 °C	[30]
	Yr10-RT-R	CAATGAACACCAGTTGTCCTA		
*Yr15*	KinⅠ-F	GGAGATAGAGCACATTACAGAC	55 °C	[31]
	KinⅠ-R	TTTCGCATCCCACCCTACTG		
*Yr17*	SC-385-F	CTGAATACAAACAGCAAACCAG	64 °C	[32]
	SC-385-R	ACAGAAAGTGATCATTTCCATC		
*Yr18*	csLV34-F	GTTGGTTAAGACTGGTGATGG	57 °C	[33]
	csLV34-R	TGCTTGCTATTGCTGAATAGT		
*Yr26*	WE173-F	GGGACAAGGGGAGTTGAAGC	55 °C	[34]
	WE173-R	GAGAGTTCCAAGCAGAACAC		
	A	GAGAAAATCAGCAGGTGG	64 °C	[35]
*YrSP*	B	GAGAAAATCAGCAGGTGC		
	C	AGCGAGTTGAGGACATTGGT		

## 3 Results

### 3.1 Seedling resistance assessment of spring wheat landraces

Two *Pst* races of CYR32 and CYR34, were used to assess the seedling resistance of 267 spring wheat landraces from 8 provinces. The aim was to clarify the resistance to stripe rust disease in these spring wheat germplasm resources. The results indicated (shown in Table 3, Table S1), there were significant differences in resistance to CYR32 and CYR34 among the tested spring wheat varieties during the seedling stage. Out of 267 spring wheat entries, 20 showed seedling resistance to CYR32 (IT = 0–6), accounting for 7.49 %, including 2 from Qinghai (Qingnong 489, Qingnong 487), 1 from Tibet (Zang 927), 1 from Sichuan (Chuan 723), 6 from Yunnan (Yun 165, Yun 254, Yun 199, Yun 458, Yun 134, Yun 437), 2 from Ningxia (Ningdi 56, Ningdi 57), and 8 from Gansu (Gan A120, Gan A130, Gan A160, Gendi 749, Gendi 659, Gendi 647, Gendi 729, Gendi 654). 33 spring wheat entries showed seedling resistance to CYR34, representing 12.35 %. Only 8 spring wheat entries, including Jinmai (Qingnong 487), Zangmuchun (Zang 927), Yigenmiao (Chuan 723), Xiaobaimaibanyi (Yun254), Baiguangtou(Gan A160), Dahonghuohe (Gandi 601), Hongguanggaungtou (Gandi 659) and Hongcuozi (Gan A120), exhibited resistance to both CYR32 and CYR34, constituting 2.99 %.

**Table 3:** Resistance identification results of wheat varieties at seedling stage and adult stage.

Resistant wheat varieties	Seedling tests			Adult-plant stage tests					
	CYR32	CYR34	CYR32 and CYR34	Xining (23XN)	Huangzhong (23HZ)	Gonghe (24 GH)	Huangzhong (24HZ)	Xining (24XN)	5 nurseries
Number	20	33	8	19	16	14	67	33	6
Percentage (%)	7.49	12.35	2.99	7.12	5.99	5.24	25.09	12.36	2.25

### 3.2 Adult plant resistance assessment of spring wheat landraces

Wheat Landraces in the natural stripe rust disease nursery of Qinghai Province, the adult plant resistance to stripe rust was assessed for 267 spring wheat landraces from 8 provinces across multiple environments over several years (23XN, 23HZ, 24XN, 24HZ, and 24 GH). The results indicated (Table 3, Table S1), in the 23XN nursery, 19 germplasms showed resistance during the adult plant stage (IT = 2), accounting for 7.12 %. In the 23HZ nursery, 16 germplasms showed resistance, representing 5.99 %. In the 24 GH nursery, 14 germplasms showed resistance, representing 5.24 %. In the 24HZ nursery, 67 germplasms showed resistance, constituting 25.09 %. In the 24XN nursery, 33 germplasms showed resistance, representing 12.36 %. Only 6 spring wheat landraces from Inner Mongolia, Yunnan, and Gansu, including Huoliaomai (Meng 1151), Dahongpao (Meng 1178), Gezama (Yun 383), Heimangmai (Yun 95), Datang Sanyuemai (Yun 207), and Banjiemang (Gandi 816), exhibited adult-plant resistance (APR) across all nurseries, accounting for 2.25 %, with Banjiemang (Gendi 816) showing immunity (IT = 0) in 2 years across 5 nurseries. Dabaipi (Meng 1164), Huakemai-2 (Yun 232), and Laomangmai (Gendi 572), these 3 spring wheat germplasms showed resistance in 4 nurseries but susceptibility in one nursery.

### 3.3 Molecular marker tests

**Fig. 1:**
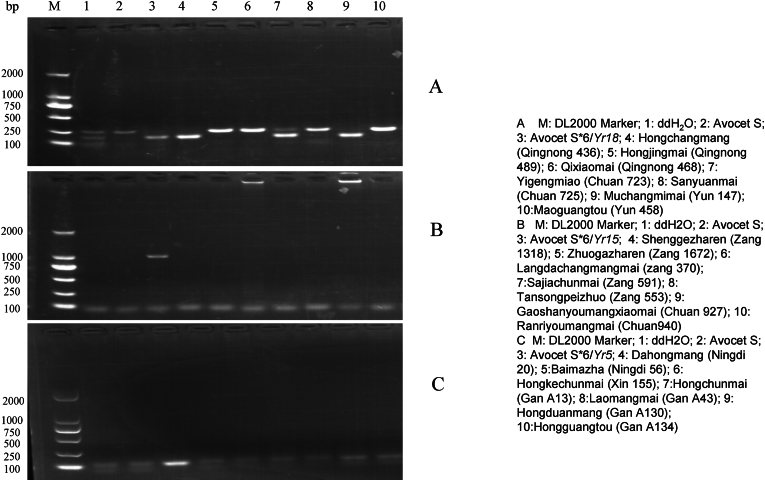
Detection results of agarose gel by using the marker csLV34 linked to the stripe rust resistance gene *Yr18* (A), the marker Kin I linked to the stripe rust resistance gene *Yr15* (B) and the marker S19M93-100 linked to the stripe rust resistance gene *Yr5* (C) in part of spring wheat landrace.

Among the 267 spring wheat germplasm resources tested (Table S1, Table 4), 79 accessions including Hongchangmang (Qingnong 436), Yigenmiao (Chuan 723), Dahongpi (Meng 1085), Mangshuibaiguangtou (Yun 223), Baimang (Ningdi 55), and Baijinbaoyin (Xin 216) et al., detected the *Yr18* (29.59 %) (Fig. 1A). 14 accessions including Kangdingxiankuaimai (Chuan 929), Xiaohongmang (Ningdi 24), and Tutoulanmai (Gan A9) et al., detected the *Yr15* (5.24 %) (Fig. 1B). 27 accessions including Datoumai (Yun 280), Shuizhaishanmai (Yun 241), Baidatou (Gan A188), and Dahonghuohe (Gandi 749) et al., molecular characterization detected the *Yr5* (10.11 %) (Fig. 1C). 3 accessions including Baidatou (Gan A203), Juezhuo-1 (Zang 1487), and Jilongxiaomai (Zang 634) detected the *Yr9* (1.12 %). 103 accessions including Dahongmai (Qingnong 418), Xiajiangqumangmai (Zang 1092), Tuanjiejizhuo (Zang 148), Gaoshanzaoshuxiaomai (Chuan 926), Huakemai-2 (Yun 232), and Qilike (Xin 135) et al., detected the *Yr10* (38.6 %). 2 accessions including Zhongfuhongmangmai (Gandi 650) and Batangmai (Yun 402) detected the *Yr17* (only 0.75 %). 56 accessions including Qinglanmai (Qingnong 505), Rendamaoyingmai (Zang 1526), Yuezuwumangmai (Chuan 883), and Gezhamai (Yun 383) et al., detected the *Yr26* (20.97 %). 23 accessions including Xiaohongmai (Qingnong 459), Gaomangmai (GanA 56), and Huoliran (Gandi 606) et al., detected the *YrSP* (8.61 %).

**Table 4:** Number of spring wheat landrace in various provinces containing stripe rust resistance genes identified with molecular markers.

Province of origin	Number of wheat cultivars							
	Yr5	Yr9	Yr10	Yr15	Yr17	Yr18	Yr26	YrSP
Qinghai	0	0	5	1	0	3	1	1
Xizang	5	2	9	0	0	0	10	3
Sichuan	3	0	12	3	0	5	3	1
Inner Mongolia	2	0	13	5	0	13	1	2
Yunnan	6	0	11	0	1	7	7	1
Ningxia	3	0	8	2	0	6	7	1
Xinjiang	0	0	6	1	0	1	2	0
Gansu	8	1	39	2	1	44	25	14
Total	27	3	103	14	2	79	56	23

60 accessions detected 2 resistance genes, the *Yr5+Yr18* (1 accession), *Yr10+YrSP* and *Yr26+YrSP* (2 accessions), *Yr18+YrSP* (3 accessions), *Yr5+Yr10*, *Yr10+Yr15* and *Yr10+Yr26* (4 accessions), *Yr5+Yr26* (5 accessions), *Yr18+Yr26* (8 accessions), and *Yr10+Yr18* (27 accessions). 24 accessions detected 3 resistance genes, with *Yr9+Yr26+YrSP*, *Yr5+Yr26+YrSP*, *Yr5+Yr10+Yr18*, *Yr10+Yr17+Yr26*, *Yr5+Yr18+YrSP* and *Yr18+Yr26+YrSP* (6 accessions), *Yr10+Yr18+YrSP* (2 accessions), *Yr5+Yr10+Yr26* (2 accessions), *Yr10+Yr18+Yr26* (6 accessions), and *Yr10+Yr15+Yr18* (8 accessions). 8 accessions carry 4 resistance genes, with 3 accessions including Gatuo (Zang 38), Xinsandaomei (Meng 1278), and Hongtuzi (Ningdi 66) detected *Yr5+Yr10+Yr26+YrSP*, Juezhuo-1 (Zang 1487) and Baidatou (GanA 203) detected *Yr5+Yr9+Yr15+Yr26*, Xiaobaimaibianyi (Yun 254) and Gaomangmai (GanA 56) detected *Yr5+Yr10+Yr18+YrSP*, and Xiaoqitou (Gandi 815) detected *Yr5+Yr10+Yr18+Yr26*. Zhongfuhongmangmai (Gandi 650) detected 5 resistance genes *Yr10+Yr17+Yr18+Yr26+YrSP*. 97 accessions did not detect any of the tested resistance genes (Table S1).

## 4 Discussion

Qinghai province is one of the most important components of the northwestern oversummering region for wheat stripe rust in China and is also the most significant base for oversummering inoculum of wheat stripe rust. In recent years, with the implementation of large-scale management in the core inoculum areas of *Pst* by the state, the amount of oversummering inoculum in the Longnan area of Gansu has gradually decreased. Due to the further expansion of winter wheat cultivation and the extensive cultivation of late-maturing spring wheat within the province, Qinghai province has become the largest oversummering area with the highest amount of inoculum for *Pst* in China in autumn [36], directly affecting the prevalence of stripe rust in Qinghai province and other wheat regions in China [37]. Therefore, understanding the resistance and distribution of resistance genes in spring wheat landraces in the Qinghai-Tibet Plateau region is instrumental in addressing the resistance of wheat varieties and the prevalence of diseases from the source, which is of great significance for the integrated control of wheat stripe rust in China.

In light of this, the current study conducted a two-year, five-location stripe rust resistance evaluation and molecular marker analysis of 267 spring wheat landraces from eight provinces. Molecular-phenotypic concordance data from Table 3, Table S1 and Fig. 1. The results indicated that seedling resistance was observed in 20 and 33 landraces accessions against CYR32 and CYR34, respectively. Further studies involving a wider range of Pst races at the seedling stage could help dissect the potential minor-effect genes or race-specific interactions in these landraces. Adult plant resistance (APR) across all test environments was demonstrated by six landraces: Huoliaomai (Meng 1151), Dahongpao (Meng 1178), Gezhamai (Yun 383), Heimangmai (Yun 95), Datangsanyuemai (Yun 207), and Banjiemang (Gandi 816). Three additional germplasms exhibited APR in four environments with susceptibility in single nurseries. These findings confirm the generally low resistance levels among evaluated landraces, particularly in Qinghai materials, aligning with previous characterizations of Qinghai-Tibet Plateau wheat germplasm [[38], [39], [40], [41], [42]]. The identified APR landraces represent valuable parental materials for enhancing genetic diversity in resistance breeding programs in this study. The APR-identified landraces (Huoliaomai, Dahongpao, Gezhamai, Heimangmai, Datangsanyuemai and Banjiemang) in this study exhibit durable, race-nonspecific resistance patterns, making them particularly valuable as parental materials for: broadening the genetic diversity of stripe rust resistance in modern breeding programs, introducing novel resistance genes/alleles absent in current elite cultivars, developing multiline varieties or gene pyramids through marker-assisted selection, serving as potential sources of both major-effect and minor-effect resistance genes. The combination of their field-validated APR performance and molecular characterization data (Table S1) suggests these landraces could significantly contribute to breeding more durable rust-resistant varieties for China spring wheat regions.

The emergence of CYR34 has significantly altered *Pst* population structures in China [43], rendering ineffective previously deployed resistance genes including *Yr10*, *Yr24/26*, and those in Guinong 22/Chuanmai 42 [44,45]. Current breeding programs rely predominantly on *Yr5* and *Yr15* for all-stage resistance, though *Yr5* virulent races have been documented [8,[46], [47], [48]], but wheat varieties containing *Yr5* have not yet been widely cultivated, and their durability of resistance has not been truly tested in production [49]. Notably, our results revealed a low representation of these genes in the germplasm from these provinces: only 10.11 % (27/267) and 5.24 % (14/267) tested positive for *Yr5* and *Yr15*, respectively. This scarcity is corroborated by nationwide surveys [[50], [51], [52], [53], [54]], suggesting limited historical utilization of these spelt-derived (*Yr5*, 2BL) [55] and wild emmer-derived (*Yr15*) genes [56,57]. Intriguingly, the 267 materials in this study are all spring wheat landraces without a background of hybridization with wheat varieties (lines) carrying *Yr5* and *Yr15*, but field susceptibility was observed in several *Yr5/Yr15* positive landraces, implying potential allelic variation or suppressor gene activity at these loci.

*Yr18*, derived from common wheat, is localized on chromosome 7DS, with Thatcher, Anza, and Jupeteco 73R among its carrier varieties. It exhibits susceptibility during the seedling stage and moderate to high resistance during the adult plant stage, and is recognized as a slow-rusting gene with durable resistance, with no physiological specialization detected to date [58]. *Yr18* was identified in 29.59 % (79/267) of accessions, confirming its broad distribution in Chinese landraces [[59], [60], [61], [62], [63]]. However, field performance variability (e.g., susceptibility in Hongchangmang (Qingnong 436) and Sanyuanmiao (Chuan 726) suggests that monogenic *Yr18* expression provides insufficient protection under high disease pressure [64]. This aligns with established models requiring cumulative minor gene effects for durable resistance [65,66]. Additionally, this study used natural disease conditions to conduct adult plant resistance assessments of the test materials in multiple environments, finding that germplasms carrying the same resistance genes or gene combinations showed varying resistance phenotypes or levels of resistance in different assessment environments, possibly influenced by other unknown pathotypes race of *Pst*, as well as environmental conditions on the Qinghai-Tibet Plateau and the timing of the assessments.

Utilizing a diverse array of resistance genes in wheat breeding is an effective approach to develop wheat varieties with durable resistance [4,67]. In this study, revealing 60 accessions detected with two-gene combinations (predominantly *Yr18*+*Yr26*). Among them, the Banjiemang (Gandi 816) carrying this combination maintained a high level of resistance in five field nurseries over two consecutive years (immune in both 2023 and 2024), exhibiting excellent disease resistance traits that warrant further research and utilization. Three-gene and four-gene combinations were detected in 24 and 8 accessions, respectively. Zhongfuhongmangmai (Gandi 650), detected 5 *Yr* genes (*Yr10+Yr17+Yr18+Yr26+YrSP*), but in two years of field adult plant resistance assessments, it only showed resistance in Huangzhong nursery (23HZ and 24HZ), while in the other three nurseries (23XN, 24XN, and 24 GH), it showed susceptibility. This may be related to the different annual and regional virulence frequencies of the locally prevalent *Pst* races under natural disease conditions, and may also be related to the differences in temperature and humidity environmental conditions during the wheat growth and pathogen infection in different years. The adult plant resistance assessment in this study in Qinghai was conducted based on natural disease occurrence, and due to the high altitude and complex topography in the Qinghai region, with significant differences in climate conditions across different areas, it has a unique stripe rust epidemic environment, leading to differences in adult plant resistance assessment results of these materials in different regions. Studies have shown that in 2011 and 2012, the virulence analysis of the *Pst* population in Qinghai Province found that some *Pst* groups collected from Huangzhong and Xining were different [68]. In the next phase, these germplasms carrying pyramided *Yr* genes will serve as parental materials for stripe rust resistance gene pyramiding breeding to achieve durable resistance through targeted gene stacking.

Breeding spring wheat varieties with resistance can reduce the range of summer hosts for stripe rust and effectively control the spread of stripe rust in western regions, even alleviating the disease conditions in eastern wheat areas. Zhao et al. [51] conducted adult plant resistance assessments on 93 local wheat germplasms from the Qinghai-Tibet spring -winter wheat regions in four nurseries in Mianyang and Chengdu, with only 10 accessions showing resistance in all nurseries. From the current situation of spring wheat resistance to stripe rust, only a very few materials such as ‘Huining 18′ have good resistance to stripe rust, and the majority have low resistance [69]. This study found only 6 spring wheat landrace accessions from Inner Mongolia, Yunnan, and Gansu that showed adult plant resistance in five nurseries over two years, indicating that most varieties have lost resistance, which is consistent with previous studies. Among them, Huiliaomai (Meng 1151) and Dahongpao (Meng 1178) from Inner Mongolia detected *Yr10+Yr15+Yr18*, and the Banjiemang (Gandi 816) from Gansu carried *Yr18+Yr26*. Gezhamai (Yun 383) from Yunnan detected the *Yr26*. Two accessions, Datangsanyuemai (Yun 207) and Heimangmai (Yun 95), did not detected the resistance genes used in this study, but showed strong resistance in field adult plant resistance assessments, suggesting that these accessions may carry other known or unknown *Yr* genes and resistance gene combinations that are resistant to the current popular races in wheat production.

In this study, the materials which do not carry resistance genes throughout the entire growth period but exhibit resistance, may potentially possess unknown antigenic resistance. Further multi-year, multi-location, and mixed-race complex identification systems are required to screen for stable resistance materials. This study found that spring wheat landrace germplasms from 8 provinces in China have poor resistance to stripe rust, and the diversity of resistance genes they carry is low. However, 6 germplasms with excellent resistance were identified, potentially carrying unknown resistance genes. Collectively, these findings provide actionable insights for optimizing stripe rust resistance breeding while highlighting critical knowledge gaps in gene-environment-pathogen interactions. Strategic utilization of landrace diversity, combined with evolutionary-informed gene deployment, remains essential for sustainable stripe rust management in China.

## 5 Conclusion

This comprehensive evaluation of 267 Chinese spring wheat landraces reveals critical insights into stripe rust resistance dynamics in northwestern epidemic regions. While overall resistance levels remain low (2.25 % showing consistent adult-plant resistance across environments), six landraces—notably Banjiemang (Gandi 816) with complete immunity. Molecular marker detected the *Yr10* (38.60 %) and *Yr18* (29.59 %) as predominant genes, with synergistic *Yr18+Yr26* combinations demonstrating durable resistance under high disease pressure. The detection of *Yr5/Yr15* in 10.11 %/5.24 % of accessions highlights underutilized genetic potential, while 97 accessions (36.33 %) lacking known *Yr* genes suggest novel resistance mechanisms requiring characterization. These findings underscore the urgent need to integrate landrace germplasm into breeding programs through marker-assisted pyramiding of *Yr18/Yr26* with undiscovered loci, coupled with regional deployment strategies accounting for *Pst* population shifts. The identified resistant accessions provide immediate resources for stabilizing wheat production in China's northwestern epidemic areas while guiding global efforts to develop climate-resilient, durable rust resistance.

## CRediT authorship contribution statement

Miaomiao Huang: Writing–original draft, data curation, investigation and conceptualization. Yuqing Zhang: Investigation, methodology, and conceptualization. Wanwei Hou: Resources, editing and conceptualization. Ping Jin and Liang Huang: Writing–review and editing, project administration, supervision, and funding acquisition.

## Funding

This study was supported by the Qinghai Provincial Natural Science Foundation for Young Scholars (No. 2022-ZJ-978Q), and the National Natural Science Foundation of China (No. 32402344).

## Declaration of competing interest

The authors declare that they have no known competing financial interests or personal relationships that could have appeared to influence the work reported in this paper.

## Data Availability

Data will be made available on request.
